# Developing synaesthesia: a primer

**DOI:** 10.3389/fnhum.2015.00211

**Published:** 2015-04-20

**Authors:** Beat Meier, Nicolas Rothen

**Affiliations:** ^1^Institute of Psychology and Center for Learning, Memory, and Cognition, University of BernBern, Switzerland; ^2^Institute of Psychology, University of SussexBrighton, UK

**Keywords:** synaesthesia/synesthesia, immune system, serotonin, GABA antagonists, development, neonatal, multisensory integration, connectivity

Synaesthesia is a variation of human experience that involves the automatic activation of unusual concurrent experiences in response to ordinary inducing stimuli. The causes for the development of synaesthesia are not well understood yet. Synaesthesia may have a genetic basis resulting in enhanced cortical connectivity during development. However, in some cases synaesthesia has a sudden onset, for example, caused by posthypnotic suggestions, drug exposure, or brain injury. Moreover, associative learning during a critical developmental period also seems to play an important role. Synaesthesia may even be acquired by training in adulthood. In this research topic, we bring together topical hypotheses, theories and empirical studies about the development of synaesthesia.

According to the *immune hypothesis* introduced by Carmichael and Simner (2013), the genes that are responsible for normal cortical development are also involved in the development of synaesthesia. As many of these genes have a function for both the immune system and for altering connectivity via axonal guidance, synaptic connectivity, and pruning, the interaction between the central nervous system and the immune system during early life may play a pivotal role in the development of synaesthesia. A common genetic basis of synaesthesia and *autism spectrum disorders* may also explain the higher prevalence of synaesthetes among patients diagnosed with autism spectrum disorder reported by Neufeld et al. (2013).

Likewise based on a neurochemical basis, Brogaard (2013) put forward the *serotonergic hyperactivity hypothesis* for the development of synaesthesia. Accordingly, excessive serotonin in the brain may be a common cause for synaesthesia induced by psychedelic drugs, brain injury, and in individuals with autism. Motivated by similar assumptions, Terhune et al. (2014) used a psychophysical approach to test this hypothesis. They found no support, neither for the serotinergic hyperactivity hypothesis nor for a related, reduced *GABA* levels hypothesis. Similarly, null findings were reported by Sinke et al. (2014) in an investigation on the relationship between *multisensory integration* and event related potentials. Nevertheless, consistent with previous research, they found evidence for alterations in early visual processing in synaesthesia.

According to the *neonatal hypothesis* synaesthetic associations between basic shapes and colors may be present already early in childhood. Brang et al. (2013) hypothesized that even when these associations can be refined by experience they can interfere with learning novel shape-color associations later. In an empirical study they found support for this hypothesis. Other determinants of synaesthetic associations include ordinality (e.g., the position in a grapheme sequence; e.g., “c” is the third letter in the alphabet) and sound. In a Japanese sample of synaesthetes, Asano and Yokosawa (2013) addressed the relative impact of ordinality, sound, and shape as potential factors that might determine the specific color of a grapheme in English and in Japanese (Hiragana). For Hiragana characters, they found that ordinality, sound and shape all contributed to the prediction of color attributes while for English only ordinality and shape were predictive. These results underline the important role of ordinality due to its primacy when learning the graphemes in childhood. Notably, ordinality can be considered as conceptual information.

Despite the notion that synaesthesia is a perceptual phenomenon, Mroczko-Wąsowicz and Nikolić (2014) emphasize that the stimuli that trigger synaesthesia (i.e., the inducers) often take the form of *concepts* and only the synaesthetic experiences (i.e., the concurrents) typically exhibit clear perceptual properties. This also holds for sequence-space synaesthesia, in which ordinal elements are experienced as occupying locations in extended areas of space. According to Price and Pearson (2013) this may very well be a variety of normal visuospatial imagery that has evolved as a mnemonic strategy to overcome developmental retardation of the phonological loop. Interestingly, an extended phenomenological investigation of spatial-form synaesthesia in a single case showed that often the synaesthetic experience followed an internal verbalisation of the inducer, indicating an auditory component to this form of synaesthesia (Gould et al., 2014). Moreover, the insights into and the kinds of synaesthetic experiences seemed to expand during the interview (cf. Price, 2014 for further considerations). These findings are in line with the *learning theory* of synaesthesia put forward by Watson et al. (2014). They emphasize the mutual influences of learning and synaesthesia and suggest that it may have evolved (and survived) *because* it is useful for learning.

A related question concerns whether synaesthesia can be induced by *training and hypnosis*. In a comprehensive review, Rothen and Meier (2014) conclude that behavioral facets of synaesthesia can be induced by training, but so far there is little evidence that this is also accompanied by a “synaesthetic” experience. In their commentary, Colizoli et al. (2014) ask for additional training studies which should be evaluated according to a diagnostic checklist. Specifically, they suggest that for each trained individual, consistency, bandwidth, automaticity, conscious experience, perceptual nature, and presence should be assessed, and potential demand effects controlled. Anderson et al. (2014) used hypnosis to investigate a potential performance advantage in a visual search task previously reported for genuine grapheme-color-synaesthetes. This did not materialize. However, accurate responses were associated with reports of more intense colors, suggesting that colors induced by posthypnotical suggestions were only perceived *after* successful target detection.

Last, but not least, two studies directly addressed the development of synaesthesia, that is, the *consistency* of inducer-concurrent pairs in grapheme-color synaesthesia across childhood development and the adult lifespan. In a longitudinal study, Simner and Bain (2013) found a protracted developmental trajectory from age 6 to age 11 with an age-related increase of the number of consistent grapheme-color associations. Meier et al. (2014) used a cross-sectional approach to investigate age-related changes in more than 400 grapheme-color synaesthetes aged 18–91. They found a decrease of the number of consistent grapheme-color association in older age. Together these findings suggest that synaesthesia follows a similar developmental trajectory as many other cognitive functions.

Overall, this Research Topic provides a comprehensive overview of different approaches available to address the development of synaesthesia. There may be large individual differences in the developmental trajectory and across different forms of synaesthesia. So far, the focus was mainly on the development of grapheme-color- and sequence-space synaesthesia which both involve cultural artifacts as inducers. Future studies should also address the development of other forms, for example, sound-color synaesthesia.

## Conflict of interest statement

The authors declare that the research was conducted in the absence of any commercial or financial relationships that could be construed as a potential conflict of interest.
